# BMRF-MI: integrative identification of protein interaction network by modeling the gene dependency

**DOI:** 10.1186/1471-2164-16-S7-S10

**Published:** 2015-06-11

**Authors:** Xu Shi, Xiao Wang, Ayesha Shajahan, Leena Hilakivi-Clarke, Robert Clarke, Jianhua Xuan

**Affiliations:** 1 Department of Electrical and Computer Engineering, Virginia Polytechnic Institute and State University, Arlington, VA, USA; 2 Departments of Oncology, Lombardi Comprehensive Cancer Center, Georgetown University, Washington, DC, USA

## Abstract

**Background:**

Identification of protein interaction network is a very important step for understanding the molecular mechanisms in cancer. Several methods have been developed to integrate protein-protein interaction (PPI) data with gene expression data for network identification. However, they often fail to model the dependency between genes in the network, which makes many important genes, especially the upstream genes, unidentified. It is necessary to develop a method to improve the network identification performance by incorporating the dependency between genes.

**Results:**

We proposed an approach for identifying protein interaction network by incorporating mutual information (MI) into a Markov random field (MRF) based framework to model the dependency between genes. MI is widely used in information theory to measure the uncertainty between random variables. Different from traditional Pearson correlation test, MI is capable of capturing both linear and non-linear relationship between random variables. Among all the existing MI estimators, we choose to use k-nearest neighbor MI (kNN-MI) estimator which is proved to have minimum bias. The estimated MI is integrated with an MRF framework to model the gene dependency in the context of network. The maximum a posterior (MAP) estimation is applied on the MRF-based model to estimate the network score. In order to reduce the computational complexity of finding the optimal network, a probabilistic searching algorithm is implemented. We further increase the robustness and reproducibility of the results by applying a non-parametric bootstrapping method to measure the confidence level of the identified genes. To evaluate the performance of the proposed method, we test the method on simulation data under different conditions. The experimental results show an improved accuracy in terms of subnetwork identification compared to existing methods. Furthermore, we applied our method onto real breast cancer patient data; the identified protein interaction network shows a close association with the recurrence of breast cancer, which is supported by functional annotation. We also show that the identified subnetworks can be used to predict the recurrence status of cancer patients by survival analysis.

**Conclusions:**

We have developed an integrated approach for protein interaction network identification, which combines Markov random field framework and mutual information to model the gene dependency in PPI network. Improvements in subnetwork identification have been demonstrated with simulation datasets compared to existing methods. We then apply our method onto breast cancer patient data to identify recurrence related subnetworks. The experiment results show that the identified genes are enriched in the pathway and functional categories relevant to progression and recurrence of breast cancer. Finally, the survival analysis based on identified subnetworks achieves a good result of classifying the recurrence status of cancer patients.

## Background

Biological systems in cancer involve multifunctional modules that coordinately regulate complex behavior [1]. Many researches focus on identifying biomarkers on high-throughput data such as DNA microarray data and RNA sequencing data. However, the high complexity of biological systems makes the single molecular approach hard to fully reveal the underlying mechanism. Integrative approaches with different data sources are needed to extract deeper insights in different levels and aspects [2]. Several methods [3-6] have been developed to integrate protein-protein interaction (PPI) data with microarray gene expression data to identify significant protein interaction networks. Chuang *et al*. [3] proposed a protein-network based approach to identify the biomarkers of metastasis within gene expression profiles. The biomarkers identified in interconnected subnetworks have shown high reproducibility and accuracy in the classification of metastatic versus non-metastatic tumors. Ideker *et al*. [5] introduced an approach to identify active subnetworks, which shows consistent condition-specific gene expression change on PPI network. The change of gene expression is measured by significance value (p-value) and further converted to z-score, then the network score can be aggregated by the z-score of the genes in the subnetwork. A simulated annealing based searching algorithm is implemented to find the maximal-scoring connected network. Chen *et al*. [6] point out that these two methods mentioned above define the network score as an aggregation of significance score of genes, which usually leave the less differentially expressed biomarkers unidentified. In order to address the concern of gene interaction, Chen *et al*. proposed a bagging Markov random field (BMRF) based method to improve the protein interaction subnetwork identification. BMRF employs maximum a posterior (MAP) principle to estimate the differential score of genes or proteins and form a novel network score that considers the pairwise gene interaction in the subnetworks. Although BMRF has achieved success in identifying biologically meaningful subnetworks, there are still some concerns about the method. BMRF treats the PPI connection equally by putting the same weight on the edges of the network, which is not true in the real case. As a cell needs to act differently in response to different stimuli, the regulatory mechanism should have condition specific preference. Accepting all the connections in the PPI database may lead to errors in MAP estimation. Furthermore, it has been proven that there are a lot of false positives in the protein interaction database [7]. Therefore, we need to quantify the dependency between genes to reduce the negative effect of false connections and improve the performance.

In this paper, we proposed an approach, namely BMRF-MI, for identifying protein interaction network by incorporating mutual information (MI) into a Markov random field (MRF) framework to model the dependency of genes. MI is developed in information theory to measure the uncertainty between random variables. As the complexity of the biological system is very high, using MI to estimate the correlation between genes can help us reveal both linear and non-linear relationships. By incorporating the quantification of the dependency between the genes, we are able to minimize the effects of false edges in protein network and identify more accurate subnetworks. We generate synthetic data to show that our method has an improved performance in protein subnetwork identification. Besides, we also apply BMRF-MI to breast cancer patient data to demonstrate the feasibility of the proposed method for real biological studies. Experimental results show that the proposed method is able to identify biologically meaningful subnetworks. Furthermore, we use survival analysis to show that the identified subnetworks can be used to predict the recurrence status of the cancer patients.

## Results and discussion

### Bagging Markov random field and mutual information (BMRF-MI) based network identification

**Figure 1:**
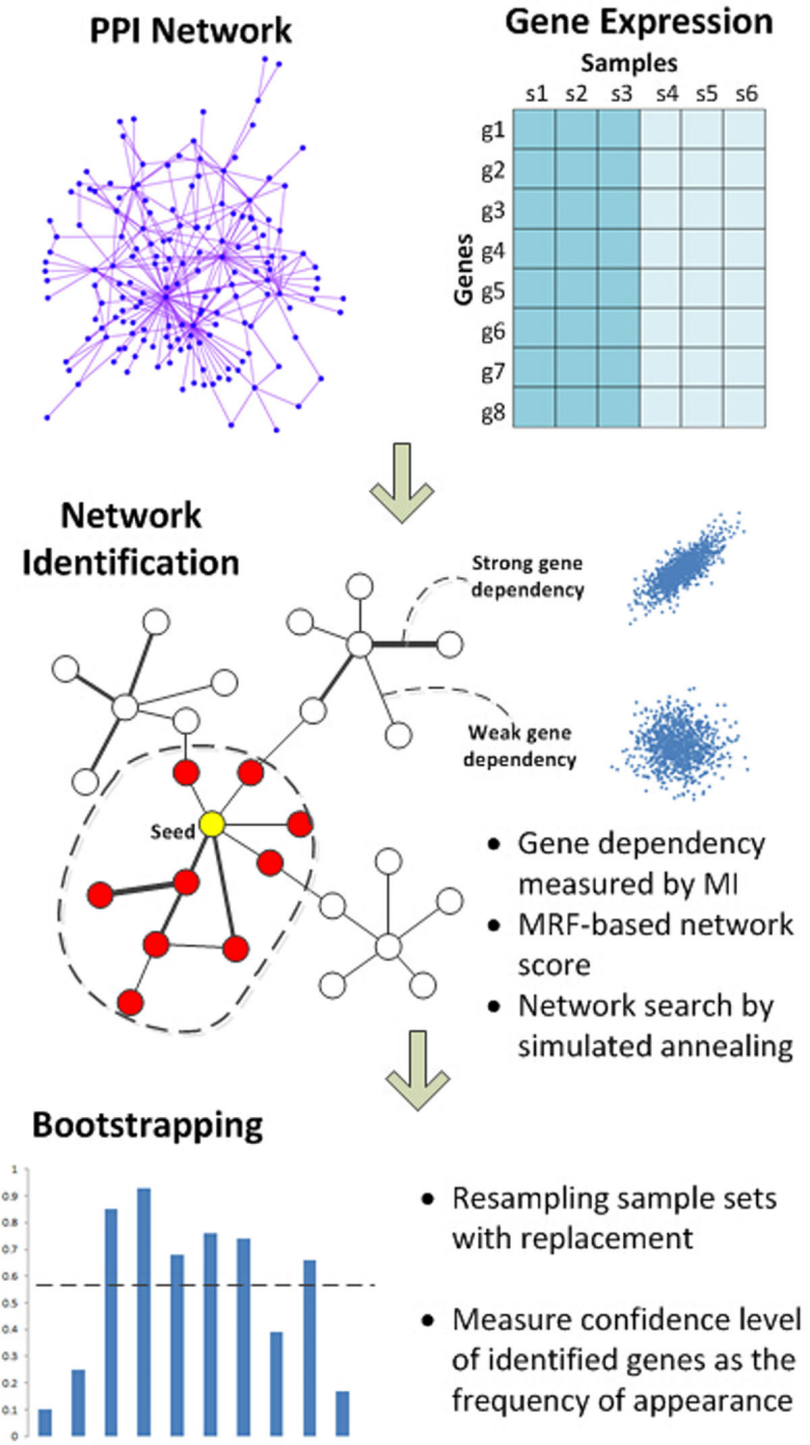
**Flow-chart of the proposed BMRF-MI approach**.

The flowchart of the proposed method is shown in Figure 1. Given the gene expression data and PPI network, we use mutual information to estimate the dependency between genes to prioritize the connections in a network. Unlike the traditional Pearson correlation test, MI measures the uncertainty between the genes, which can capture more complex relationships. We model the gene dependency in a network by integrating MRF with MI, then the network score is formed by MAP estimation. Considering the computational complexity, we applied simulated annealing searching algorithm to find the optimal network. The search starts from several selected genes and grows to construct a network with a maximum network score. We then apply a non-parametric bootstrapping to improve the robustness and reproducibility of the method. A number of sample sets can be generated by randomly sampling samples with a replacement. The confidence level of identified genes can be measured as the frequency of appearance on the sampled data sets. The final network is composed of the genes with high confidence level.

### Simulation experiments

The simulation PPI network is an estrogen receptor (ER) related PPI network with 376 nodes and 1,825 edges extracted from the HPRD database [6,8]. The ground truth subnetwork is constructed by the genes in pathways closely related to breast cancer, which has 35 genes and 89 interactions. The dependency between genes can be constructed as a symmetric matrix, and the magnitude of the element indicates the strength of dependency. The genes in the ground truth subnetwork are set to have a higher dependency value, which means that they have stronger mutual dependency than the other genes. We use the Markov random field model developed by Wei *et al*. [9] to simulate the differential state of genes. Based on the differential state and gene dependency, the gene expression data is simulated from multivariate Gaussian distribution with 40 samples in each phenotype (80 samples in total), which takes the dependency matrix as the covariance matrix. For differential genes, a random difference will be generated to differentiate the mean level of two phenotypes. For non-differential genes, the gene expression data comes from the same distribution. The differential z-score is calculated by inverse cumulative standard normal distribution of p-value estimated from Student's t-test. The false positive rate (FPR) of the simulation data can be controlled by a weight parameter *w *in the MRF model introduced by Chen *et al*. [6]. We simulate the data by varying the FPR ranging from 30% to 85% to evaluate the performance of identifying differential expressed protein interaction networks under different levels.

**Figure 2:**
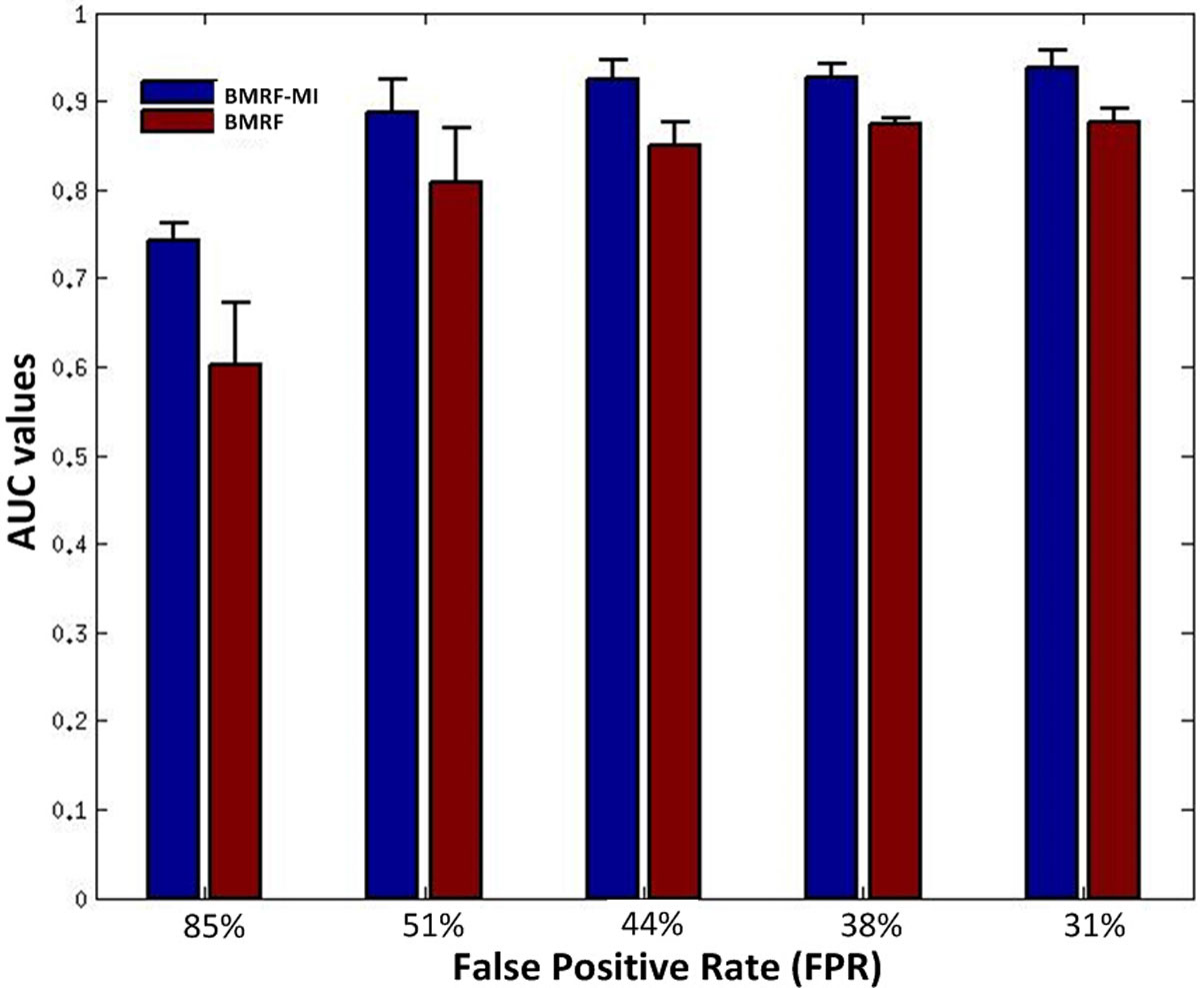
**AUC values of BMRF-MI and BMRF on simulation data under different conditions**.

We first use the area under the receiver operating characteristic (ROC) curve (AUC) as a criterion to evaluate the performance of BMRF-MI and BMRF in terms of network identification accuracy. By applying a bootstrapping strategy, BMRF-MI and BMRF will assess the confidence level of the genes in the identified network. Then different threshold of confidence level can be set to calculate the sensitivity and specificity so as to obtain the ROC curve. For each method, 5 different simulation data are tested to address the concern of variance of the performance. Figure 2 shows the AUC value of the three methods and the detail values are shown in Table 1. It can be seen that BMRF-MI performs better than BMRF, especially when the FPR is high. Under high FPR condition, BMRF-MI is able to use the dependency between the genes to correct the differential state of the genes. Although BMRF also considers the pairwise gene dependency relationship in network, the connection between genes are not differentiated, which will make the differential state of some genes affected by unrelated genes. As a result, BMRF-MI can achieve an increase of 0.05 to 0.14 in AUC under different conditions.

**Table 1:** AUC values for subnetwork identification.

FPR	BMRF-MI	BMRF
31%	**0.9376**	0.8768
38%	**0.9255**	0.8734
44%	**0.9252**	0.8496
51%	**0.8871**	0.8082
85%	**0.7430**	0.6015

**Figure 3:**
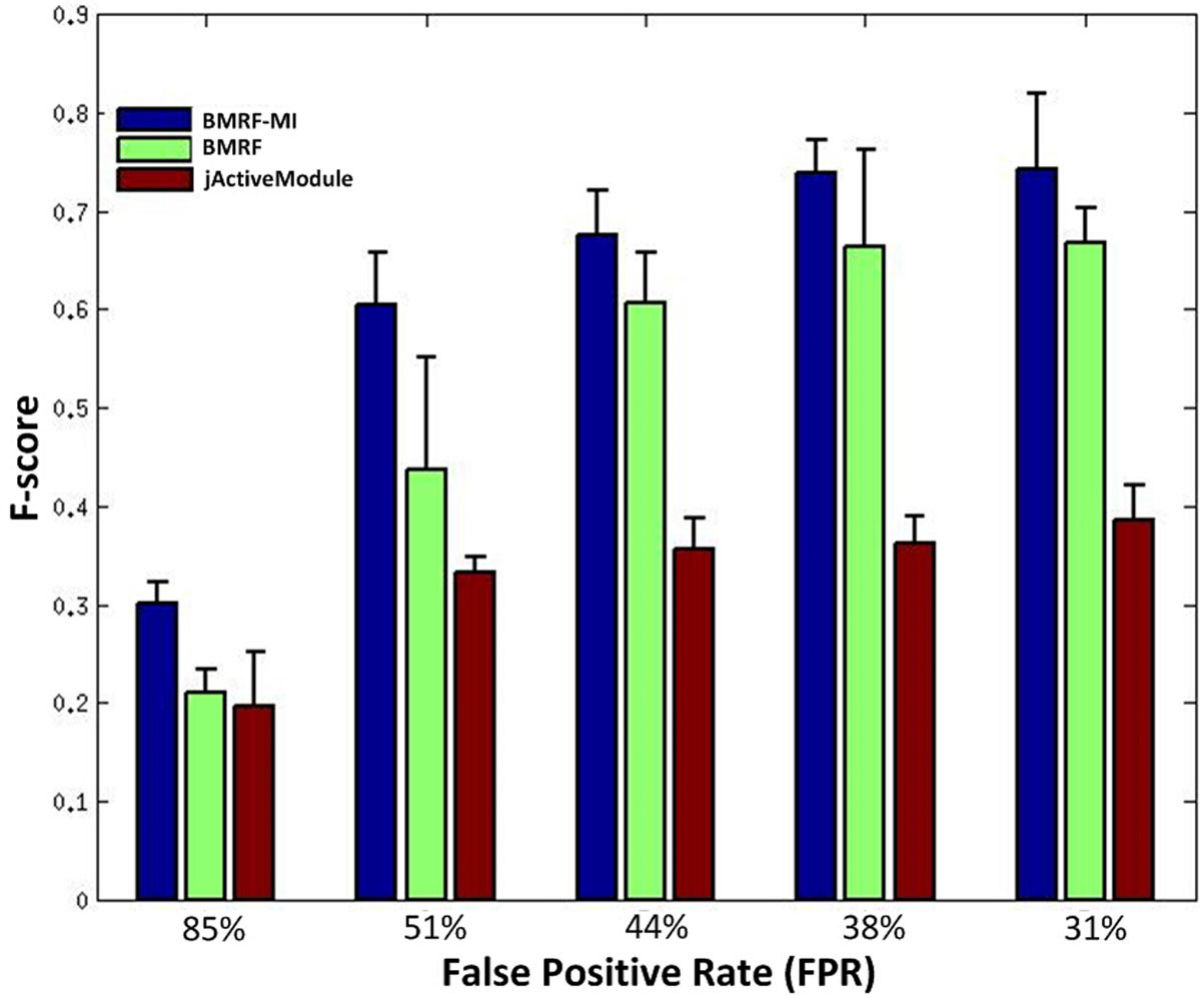
**F-scores of BMRF-MI and competitive methods on simulation data under different conditions**.

Besides BMRF, one popular method, jActiveModules proposed in [5], is selected to compare the performance. As jActiveModules does not prioritize genes in the subnetwork, we cannot use AUC to evaluate the performance. Here we use F-score (calculated as 2 * (precision * recall) / (precision + recall)) as a metric to comprehensively assess the subnetwork identification accuracy. Figure 3 and Table 2 show the F-score of the three methods. It can be seen that BMRF-MI outperforms competing methods with an average improvement of 0.09 in F-score under different conditions. As described in [5], jActiveModule only identifies the subnetwork with optimal aggregated z-score of genes, thus it will identify a lot of false positive genes in the network, which leads to low F-score.

**Table 2:** F-scores for subnetwork identification.

FPR	BMRF-MI	BMRF	jActiveModule
31%	**0.7424 **	0.6674	0.3862
38%	**0.7388**	0.6649	0.3627
44%	**0.6763**	0.6079	0.3558
51%	**0.6048 **	0.4371	0.3324
85%	**0.3010**	0.2145	0.1965

### Breast cancer microarray data

We further tested our method on one estrogen receptor (ER) related breast cancer patient dataset introduced in Loi *et al*. [10] to identify subnetworks related to recurrence of breast cancer. The patients samples are divided into 'early recurrence' group (≤ 5 years) and 'late recurrence' group (> 5 years) based on survival time. We finally got 19 samples in 'early recurrence' group and 28 samples in 'late recurrence' group. The PPI network data is obtained from HPRD database [8], which contains about 9000 genes and 35000 interactions. We further extracted an ER focused PPI network with 2545 genes and 15094 connections by finding the subnetwork within two jumps from ER. The 199 seed genes are selected from the ER focused network with node degree larger than 10. For the differential score of the genes, we perform Student's t-test on the 2545 genes between two groups of samples to calculate the p-value and convert the p-value to z-score by inverse cumulative standard normal distribution. The gene dependency is estimated by R package 'parmigene'[11], which implements the kNN-MI estimator mentioned in Kraskov *et al*. [12]. For the bootstrapping process, the confidence level threshold is set to 0.3 to find significant genes in network.

**Figure 4:**
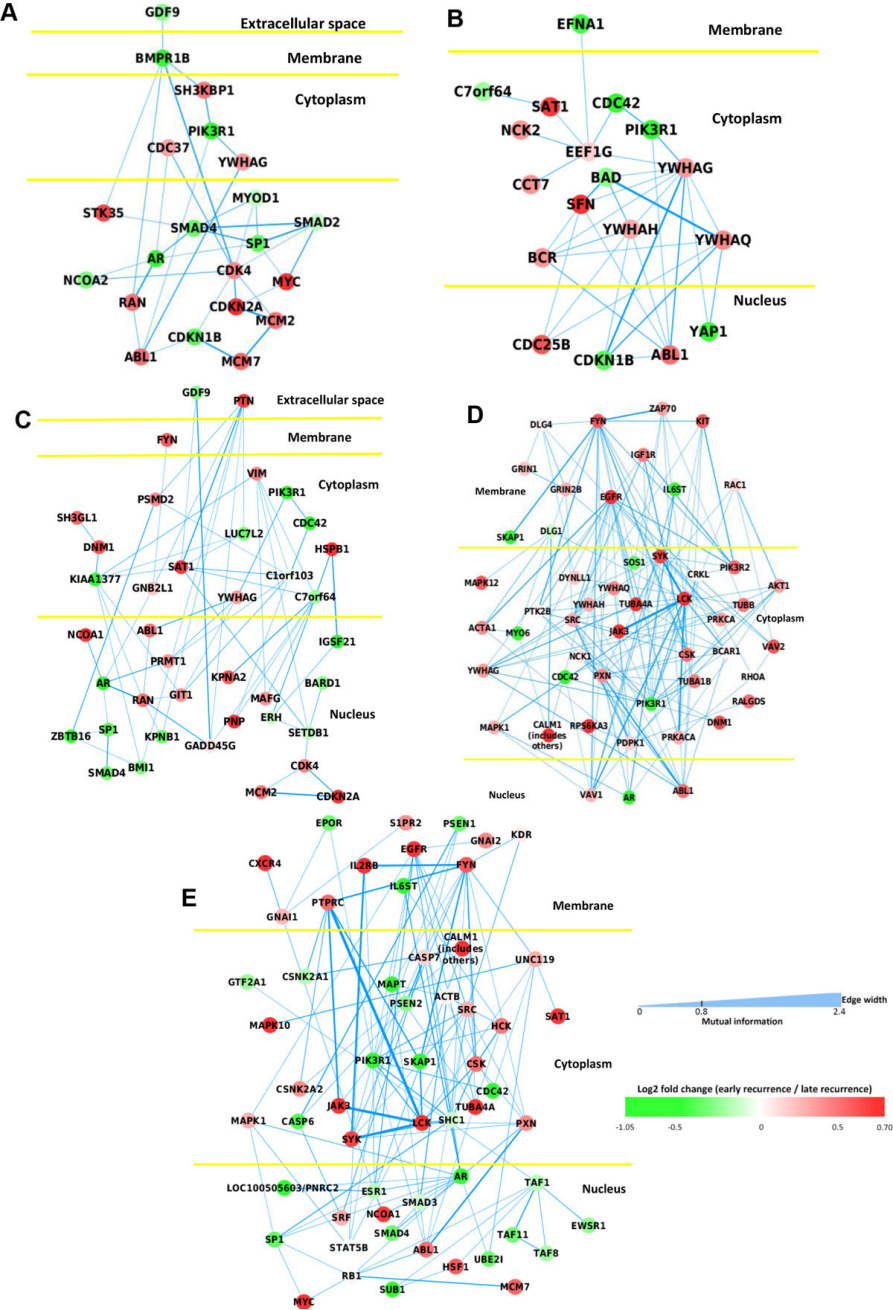
**Identified subnetworks and functional annotations**: (A) Net1: Cell Cycle: 4.40e-12, TGF-Beta signaling pathway: 7e-5; (B) Net2: Cell Cycle: 2.90e-7, ErbB signaling pathway: 5.10e-5; (C) Net3: Cell Cycle: 5.70e-6; (D) Net4: ErbB signaling pathway: 2.00e-9, Insulin signaling pathway: 1.90e-5, MAPK signaling pathway: 8.40e-5; and (E) Net5: ErbB signaling pathway: 9.70e-7.

Among the 199 identified subnetwork, we select 33 significant subnetworks with size larger than 10 and score larger than 2.576 (corresponding to p-value 0.005). Then we applied affinity propagation clustering (APC) [13] on the significant subnetworks (see Methods for detail) to merge related networks. We use the default setting of APC and get 5 subnetworks (Net1-5), which are shown in Figure 4. The color of the node shows the log2 fold change of the gene; the red genes express higher in 'early recurrence' group and the green genes express higher in 'late recurrence' group. To study the function of these networks, we applied DAVID functional annotation tool [14] to analyze the genes. The identified networks are significantly enriched in breast cancer related pathways including Cell cycle (supported by Net1, 2 and 3), TGF-Beta signaling pathway (supported by Net1), ErbB signaling pathway (supported by Net2, 4 and 5), Insulin signaling pathway (supported by Net4) and MAPK signaling pathway (supported by Net4). The details of the functional annotation results are shown in Table 3. Beside the lists of genes, we also show the dependency between genes by the thickness of edges in the network. For example, in Net1, we see that the cyclin-dependent kinases (CDK) genes are strongly connected with the minichromosome maintenance (MCM) genes. Johnson *et al*. [15] show that the CDK inhibitors will lead to increased MCM complex association with DNA and MCM is closely with DNA damage [16], which is related to breast cancer recurrence. In addition, Net2 shows strong connection between BAD and YWHAQ, which is associated with cell death and validated by [17]. Moreover, the genes in common pathway tend to have strong mutual dependency. For example, it can be seen from Net2 that the cell cycle genes including YWHAG, YWHAQ, YWHAH, CDKN1B and ABL1 are closely connected.

**Table 3:** P-values of the enriched pathways of identified subnetworks from functional annotation.

Identified Pathway	Net1	Net2	Net3	Net4	Net5
Cell Cycle	4.40e-12	2.90e-7	5.70e-6	-	-
TGF-Beta signaling	7.00e-5	-	-	-	-
ErbB signaling	-	5.10e-5	-	2.00e-9	9.70e-7
Insulin signaling	-	-	-	1.90e-5	-
MAPK signaling	-	-	-	8.40e-5	-

**Figure 5:**
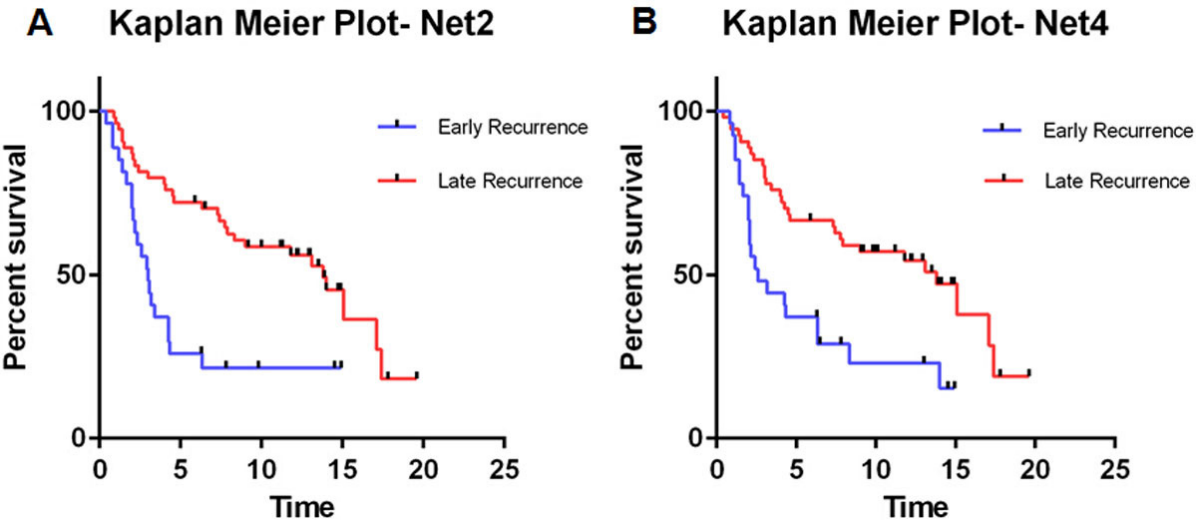
**Kaplan-Meier curve of independent test on in-house data**: (A) Net2: log-rank test p-value 0.0002; and (B) Net4: log-rank test p-value 0.0009.

In order to show the significance of the subnetworks identified from Loi et al. data, we train the netSVM [18] classifier to classify breast cancer patients from our in-house data into 'early recurrence' (≤ 5 years) group and 'late recurrence' (> 5 years) group. Our in-house data set consists of 81 samples which can be divided into 35 'early recurrence' samples and 46 'late recurrence' samples. netSVM is a network based classifier specially designed for cancer prediction by integrating gene expression data and protein interaction data. For each subnetwork, we use 5-fold cross-validation to train netSVM classifier on Loi et al. data and independently test on our in-house data. The prediction results are further analyzed by Kaplan-Meier survival analysis. Due to the heterogeneity of cancer samples, it is very hard to validate all identified subnetworks on other datasets. Among all the five subnetworks, Net2 and Net4 are significant in differentiating the survival curve of two groups with log-rank test p-value of 0.0002 and 0.0009, which is shown in Figure 5.

## Conclusion

In this paper, we have proposed a new method by incorporating mutual information into a Markov random field based framework to tackle the problem of protein interaction subnetwork identification. The proposed method is tested by simulation data with different experimental conditions. We have observed significant improvements in terms of the accuracy of subnetwork identification. To validate the efficacy of the method in real biomedical applications, a breast cancer patient dataset is used for the identification of protein interaction networks related to recurrence of breast cancer. The identified subnetworks are significantly enriched in pathways related to the progression and recurrence of breast cancer. We further validate the significance of the subnetworks on other dataset by predicting the recurrence status of patients.

## Methods

### Gene dependency estimation

Inferring the gene expression dependency is very important to network identification due to the complex regulatory mechanisms existed in biological system. In this paper, we use mutual information as a measure of the dependence of gene expression. Mutual information is widely applied in information theory to detect the relationship between random variables; and it is capable to capture both linear and non-linear relationship. The mutual information between two gene expression × and Y is defined as:

(1) $$I\left(X,Y\right)=\int \int p\left(x,y\right)\text{log}\frac{p\left(x,y\right)}{p_{X}\left(x\right)p_{Y}\left(y\right)}dxdy$$

where $p_{X}\left(x\right)=\int p\left(x,y\right)dy$ and $p_{Y}\left(y\right)=\int p\left(x,y\right)dx$ are the marginal distributions of × and Y respectively. As the analytical expression of $p\left(x,y\right),p_{X}\left(x\right)$ and *p_Y_*(*y*) are intractable, discretization is one intuitive solution which is usually applied to gene expression data to approximate the distributions. After partitioning both × and Y into a finite number of bins, all the probabilities can be calculated by counting the number of points falling into corresponding bins. Then Equation (1) can be approximated as:

(2) $$I_{bin}\left(X,Y\right)=\underset{\left(x,y\right)\in bin}{\sum }p\left(x,y\right)\text{log}\frac{p\left(x,y\right)}{p_{X}\left(x\right)p_{Y}\left(y\right)}.$$

However, this method depends on the way of discretizing bins, which will lead to errors of estimation. To achieve a better estimation of mutual information, Kraskov *et al*. [12] proposed a k-nearest neighbor MI (kNN-MI) estimator. The kNN-MI estimator utilizes the distance between the point and its kth nearest neighbor to estimate the mutual information:

(3) $$I_{kNN}\left(X,Y\right)=\psi \left(k\right)-\frac{1}{k}-<\psi \left(n_{x}\right)+\psi \left(n_{y}\right)>+\psi \left(N\right),$$

where $\psi \left(x\right)=\Gamma {\left(x\right)}^{-1}\frac{d\Gamma \left(x\right)}{dx}$ is the digamma function, $<f\left(x\right)>=\frac{1}{\text{N}}E\left[f\left(x\right)\left(i\right)\right]$ averages *f*(*x*) all over *i *and realizations, and *N *is the number of realizations or samples. Assume $\in_{X}\left(i\right)$ and $\in_{Y}\left(i\right)$ are the distance from point *i *with coordinate (x_i_,y_i_) to its k-th nearest neighbor in subspace × and Y respectively, *n_x _*and *n_y _*are the number of points in the set $\left\{\left(x_{j},y_{j}\right)|{||x}_{j}-x_{i}||\leq \frac{\in_{\text{x}}\left(i\right)}{2}\;\text{and}\;||y_{j}-y_{i}||\leq \frac{\in_{y}\left(i\right)}{2}\right\}$. Sales *et al*. [11] compare kNN-MI estimator with several other mutual information estimators such as KDEMI [19], the Miller-Madow [20] and the Schurmann-Grassberger estimators [21] and show that the kNN-MI estimator has the minimum bias. In this paper, we use the 'parmigene' [11], a well-developed R package, to estimate the mutual information between all pair of genes. The mutual information can be represented as a symmetric matrix **W**, where *w*(*i, j*) is the estimated mutual information between the *ith *and the *jth *gene.

### Markov random field (MRF) based network score

In order to incorporate the gene dependency in the model, the network score is defined based on MRF framework. Given a subnetwork M with m genes, we can define a multivariate random variable $f={\left[{\text{f}}_{1},\ldots {\text{f}}_{\text{m}}\right]}^{\text{T}}$ as the discriminative score of the m genes between two phenotypes. In the context of PPI network, we assume that the discriminative score forms a Markov random field. For a given gene *i *in the subnetwork, let *N_i _*represents the number of genes connected with gene *i*. By the virtue of Markov property, we can assume that the discriminative score of gene *i *depends on the discriminative scores of its *N_i _*neighbor genes:

(4) $$\text{P}(f_{i}\text{|}f_{S-\left\{i\right\}})=\text{P}(f_{i}\text{|}f_{N_{i}}),\text{P}(f)\geq 0,$$

where S is the set of all the genes in the PPI network. We can then use Gibbs distribution to specify the joint probability of **f**:

(5) $$\text{P}\left(f\right)=\frac{1}{\text{K}}e^{-\frac{1}{T}U\left(\text{f}\right)},$$

where K is a normalization constant to guarantee the total probability as 1, *T *is a temperature parameter that controls the sharpness of the distribution and *U*(***f***) is the prior energy function over 1-vertex and 2-vertex cliques of the subnetwork. The 1-vertex clique *C*_1 _is defined as the whole gene set and 2-vertex clique *C*_2 _is defined as the set of neighbor genes on *C*_1_. *U*(***f***) proposed by Chen *et al*.[6] can be represented by the sum of clique potential *V_c_*(**f**):

(6) $$U\left(\text{f}\right)=V_{c_{1}}\left(\text{f}\right)+V_{c_{2}}\left(\text{f}\right)=-\frac{1}{\text{m}}\underset{i\in C_{1}}{\sum }f_{i}+\frac{\lambda }{k}\underset{i,j\in C_{2}}{\sum }{\left(\frac{f_{i}}{\sqrt{d_{i}}}-\frac{f_{j}}{\sqrt{d_{j}}}\right)}^{2},$$

where m is the number of genes, *k *is the number of edges, λ is a trade-off parameter and *d_i _*is the node degree of gene *i*. The first term in the equation estimates discriminative level of the network and the second term accounts for the smoothness of the discriminative score. As seen from Equation (6), smaller U(**f**) will lead to a more probable network configuration, thus identifying a network with genes that have high and consistent discriminative score is our goal. However, there are some concerns needed to be addressed. In the first term, the average of discriminative score tends to decrease the variance of **f **when the number of genes increases, which makes the network score not comparable over different sizes of networks. In order to make the variance comparable, we modified the first term by changing the coefficient $\frac{1}{\text{m}}$ to $\frac{1}{\sqrt{m}}$. The second term utilizes the MRF framework to smooth the discriminative scores of the genes in a network; however, this term treats each connection equally. Here we incorporate the gene dependency estimated from kNN-MI:

(7) $$U\left(\text{f}\right)=V_{c_{1}}\left(\text{f}\right)+V_{c_{2}}\left(\text{f}\right)=-\frac{1}{\sqrt{m}}\underset{i\in C_{1}}{\sum }f_{i}+\frac{\lambda }{k}\underset{i,j\in C_{2}}{\sum }{\left(\frac{f_{i}}{\sqrt{d_{i}}}-\frac{f_{j}}{\sqrt{d_{j}}}\right)}^{2}w\left(i,j\right),$$

where *w*(*i, j*) is the mutual information between *ith *gene and *jth *gene and the node degree *d_i _*can be calculated as $\underset{\left\{j|\left(i,j\right)\in C_{2}\right\}}{\sum }w\left(i,j\right)$. The weights added in the second term of Equation (7) can guarantee the smoothness of the discriminative scores over genes with strong dependency.

Due to the large noise existed in the expression data, **f **cannot be directly estimated. In order to lessen the effect of noise and address the concern of gene dependency, MAP estimation method is applied on the observed score to estimate **f**. Suppose the observed discriminative scores are$z={\left[z_{1},\ldots ,z_{m}\right]}^{T}$. Here the z-score *z_i _*can be calculated from corresponding p-value *p_i _*by the inverse Gaussian cumulative density function. In this method, p-value is calculated by Student's t-test between two phenotypes. To account for the noise in the gene expression data, we assume that

(8) $$z=f+e,e\sim \text{N}\left(0,I\right),$$

where **e **is Gaussian noise and **I **is the identity matrix. Given the z-score, we can estimate the discriminative score $\hat{f}$ by MAP estimation. Based on Bayes's rule and Gibbs distribution, the estimation can be expressed as:

(9) $$\hat{f}=\underset{f}{\text{arg min}}\left(U\left(\text{f}\right)+U\left(\text{z}|\text{f}\right)\right),$$

where U(**f**) is specified in Equation (7). U(**z**|**f**) is the likelihood potential, which can be derived from the distribution of **e **as:

(10) $$\text{U}\left(z|f\right)=\frac{\gamma }{\text{m}}\overset{m}{\underset{i=1}{\sum }}{\left(z_{i}-f_{i}\right)}^{2},$$

where m is the number of genes in a network and γ is a trade-off parameter. With the estimator $\hat{f}$, the network score can be defined as:

(11) $$\text{Score}\left(\text{M}\right)=\frac{1}{\sqrt{\text{m}}}\overset{m}{\underset{i=1}{\sum }}{\hat{f}}_{i}-\frac{\lambda }{k}\underset{i,j\in C_{2}}{\sum }{\left(\frac{{\hat{f}}_{i}}{\sqrt{d_{i}}}-\frac{{\hat{f}}_{j}}{\sqrt{d_{j}}}\right)}^{2}w\left(i,j\right)-\frac{\gamma }{m}\overset{m}{\underset{i=1}{\sum }}{\left(z_{i}-{\hat{f}}_{i}\right)}^{2}.$$

Equation (11) provides a good measurement of network score with consistent mean and variance on networks with different sizes under the assumption that **f **has zero mean; however, the assumption usually violates in the real application. Here we estimate the background distribution of network score with different sizes by random sampling subnetworks from the whole PPI network. The network score can be normalized by:

(12) $$\text{Scor}{\text{e}}_{\text{norm}}\left(\text{M}\right)=\frac{Score\left(M\right)-\mu \left(M\right)}{\sigma \left(M\right)},$$

where *μ*(*M*) and *σ*(*M*) are the mean and standard deviation estimated from the null distribution of the networks with the same size of M.

### Simulated annealing searching

Given the network score definition, finding the optimal network with the highest network score is an NP hard problem. Instead of using exhausted searching approach, a probabilistic approach for global optimization, simulated annealing, is applied here. To reduce the computational complexity, we start the simulated annealing searching from 'seed' genes, which are pre-selected from the PPI network. Several constraints are made to further increase the network searching efficiency: (i) the searching space is restricted to a local 2-jump network from 'seed' node and (ii) the searching will be terminated when no significant improvement is observed.

### Confidence level measurement

Due to the large noise of data and heterogeneity of samples, the reproducibility of subnetwork identification is usually low. Furthermore, the number of samples is usually limited in biological experiment due to cost and quality issue, which will introduce bias to the results. In order to get more robust results, we applied a non-parametric bootstrapping strategy to measure the confidence level of the genes in the identified network. For each bootstrap, we generate a data set by randomly sampling the samples from the gene expression data with replacement. Applying BMRF-MI on the generated data sets, we can measure the confidence level of genes as the frequency of appearance. We are more confident about the genes with high frequency; then a threshold can be set to generate the final network.

### Clustering networks by affinity propagation clustering (APC)

The identified subnetworks from BMRF-MI are merged by affinity propagation clustering (APC) [13] to generate more comprehensive biologically meaningful network. APC clusters the data points by passing the messages which include 'responsibility' and 'availability' between them. The 'responsibility' and 'availability' together indicate how appropriate to choose the points to be the 'exemplar' of the cluster. The similarity between two networks is defined as:

(13) $$\text{S}(i,j)=2\times \frac{\#\;of\;genes\;in\;both\;Net\;i\;and\;Net\;j}{\#\;of\;genes\;in\;Net\;i+\#\;of\;genes\;in\;Net\;j}.$$

S(*i, j*) has a value between 0 and 1 and higher value indicates higher similarity between the two networks. The number of exemplars or clusters can be automatically determined without pre-configuration, which is different from traditional clustering methods such as k-means clustering.

## Competing interests

The authors declare that they have no competing interests.

## Authors' contributions

JX, XS and XW designed the framework of the proposed method. XS and XW constructed and implemented the method, and performed simulation experiments. JX designed the breast cancer study, and XS performed the data analysis with the help from XW. AS, LHC and RC provided their biological interpretations on the breast cancer results. XS and JX wrote and revised the manuscript. All authors read and approved the final manuscript.

## Declarations

The publication costs for this article were funded by National Institutes of Health (NIH) [CA149653].

This article has been published as part of *BMC Genomics *Volume 16 Supplement 7, 2015: Selected articles from The International Conference on Intelligent Biology and Medicine (ICIBM) 2014: Genomics. The full contents of the supplement are available online at http://www.biomedcentral.com/bmcgenomics/supplements/16/S7.
